# Aqua­chlorido{6,6′-dimeth­oxy-2,2′-[ethane-1,2-diylbis(nitrilo­methanylyl­idene)]diphenolato-κ^2^ *O* ^1^,*N*,*N*′,*O* ^1′^}cobalt(III) dimethyl­formamide monosolvate

**DOI:** 10.1107/S1600536812011324

**Published:** 2012-03-21

**Authors:** Yun Wei, Ting Pang, Jiacheng Liu, Meng Li, Lili Liang

**Affiliations:** a College of Chemistry and Chemical Engineering, Key Laboratory of Eco-Environment-Related Polymer Materials of the Ministry of Education, Key Laboratory of Polymer Materials of Gansu Province, Key Laboratory of Bioelectrochemistry and Environmental Analysis of Gansu, Northwest Normal University, Lanzhou 730070, People’s Republic of China

## Abstract

In the title compound, [Co(C_18_H_18_N_2_O_4_)Cl(H_2_O)]·C_3_H_7_NO, the Co^III^ ion is six-coordinated by a tetra­dentate 6,6′-dimeth­oxy-2,2′-[ethane-1,2-diylbis(nitrilo­methanylyl­idene)]diphenolate ligand, with a chloride ion and an aqua ligand in the apical positions. The compound crystallized as a dimethyl­formamide (DMF) monosolvate. In the crystal, complex mol­ecules are linked *via* O—H_water_⋯O hydrogen bonds to form a dimer-like arrangement. These dimers are linked *via* a C—H⋯Cl inter­action, and the DMF mol­ecule is linked to the complex mol­ecule by C—H⋯O inter­actions.

## Related literature

For related literature on metal complexes of Schiff bases, see: Aurangzeb *et al.* (1994 ▶); Hulme *et al.* (1997 ▶); Li *et al.* (2008 ▶); Wang *et al.* (1979 ▶); Xing (2009 ▶). For transition metal complexes of Schiff bases derived from *o*-vanillin, with anti­bacterial activity, see: Liu *et al.* (1990 ▶); Viswanathamurthi *et al.* (2000 ▶); Yeap *et al.* (2003 ▶). For the crystal structure of the ligand, see: Xia *et al.* (2006 ▶). For the crystal structure of the monohydrate form of the title complex, see: Xing (2009 ▶).
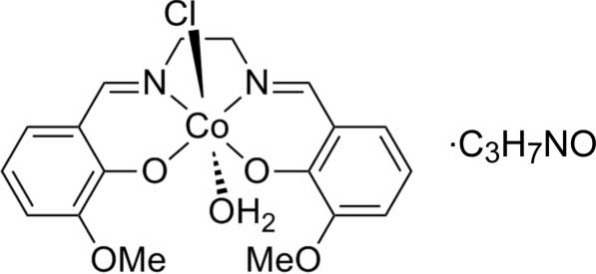


## Experimental

### 

#### Crystal data

[Co(C_18_H_18_N_2_O_4_)Cl(H_2_O)]·C_3_H_7_NO
*M* *_r_* = 511.84
Monoclinic, 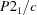

*a* = 13.1384 (13) Å
*b* = 13.3144 (19) Å
*c* = 14.0120 (9) Å
β = 110.198 (6)°
*V* = 2300.4 (4) Å^3^
*Z* = 4
Mo *K*α radiation
μ = 0.90 mm^−1^
*T* = 293 K
0.24 × 0.22 × 0.20 mm

#### Data collection

Bruker APEXII CCD diffractometer
Absorption correction: multi-scan (*SADABS*; Sheldrick, 2003 ▶) *T*_min_ = 0.812, *T*_max_ = 0.840
12275 measured reflections
4034 independent reflections
2827 reflections with *I* > 2σ(*I*)
*R*_int_ = 0.087

#### Refinement

*R*[*F* ^2^ > 2σ(*F* ^2^)] = 0.058
*wR*(*F* ^2^) = 0.140
*S* = 1.01
4034 reflections
293 parameters
H-atom parameters constrained
Δρ_max_ = 0.70 e Å^−3^
Δρ_min_ = −0.39 e Å^−3^

### 

Data collection: *APEX2* (Bruker, 2008 ▶); cell refinement: *SAINT* (Bruker, 2008 ▶); data reduction: *SAINT*; program(s) used to solve structure: *SHELXS97* (Sheldrick, 2008 ▶); program(s) used to refine structure: *SHELXL97* (Sheldrick, 2008 ▶); molecular graphics: *SHELXTL* (Sheldrick, 2008 ▶); software used to prepare material for publication: *SHELXTL*.

## Supplementary Material

Crystal structure: contains datablock(s) I, global. DOI: 10.1107/S1600536812011324/su2386sup1.cif

Structure factors: contains datablock(s) I. DOI: 10.1107/S1600536812011324/su2386Isup2.hkl

Additional supplementary materials:  crystallographic information; 3D view; checkCIF report

## Figures and Tables

**Table 1:** Hydrogen-bond geometry (Å, °)

*D*—H⋯*A*	*D*—H	H⋯*A*	*D*⋯*A*	*D*—H⋯*A*
O5—H5*A*⋯O2^i^	0.96	1.98	2.830 (3)	146
O5—H5*A*⋯O4^i^	0.96	2.22	2.964 (4)	134
O5—H5*B*⋯O1^i^	0.96	2.11	2.840 (3)	131
O5—H5*B*⋯O3^i^	0.96	1.97	2.854 (3)	151
C9—H9*B*⋯O6^ii^	0.97	2.47	3.355 (7)	152
C10—H10⋯O6^ii^	0.93	2.56	3.287 (7)	135
C17—H17*A*⋯Cl5^iii^	0.96	2.79	3.744 (4)	175

Symmetry codes: (i) 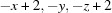; (ii) 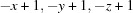; (iii) 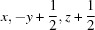.

**Fig. 1.**
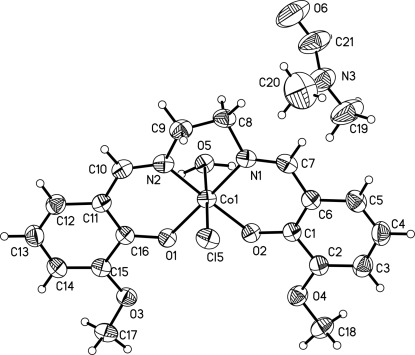
A view of the molecular structure of the title complex, with the atom numbering scheme. Displacement ellipsoids are drawn at the 50% probability level.

## supplementary crystallographic information

### Comment

Polydentate Schiff base ligands and their metal complexes have been studied for decades (Aurangzeb *et al.*, 1994, Hulme *et al.*, 1997; Li *et al.*, 2008; Wang *et al.*, 1979; Xing, 2009). Transition metal complexes of Schiff bases derived from *o*-vanillin have attracted more attention during past years due to their antibacterial activity (Liu *et al.*, 1990; Viswanathamurthi *et al.*, 2000; Yeap *et al.*, 2003). Herein, we report on the synthesis and crystal structure of the title cobalt(III) complex.

The molecular structure of the title compound is illustrated in Fig. 1. The Co^III^ ion is coordinated to two N and two O atoms of the tetradentate 6,6'-dimethoxy-2,2'-[ethane-1,2-diylbis(nitrilomethanylylidene)]diphenolate ligand, a Cl^-^ ion and one water molecule. The compound crystallized with a molecule of dimethylformamide, used as solvent. The crystal structure of the ligand has been reported previously (Xia *et al.*, 2006), as has the monohydrate form of the title complex (Xing, 2009). The Cl5—Co1—O5 bond angle is 178.77 (7)° suggesting that the Co^III^ ion has a slightly distorted octahedral environment, with atoms N1, N2, O1 and O2 occupying the equatorial positions, while atoms Cl5 and O5_water_ occupy the axial positions.

In the crystal, complex molecules are linked via O-H_water_···O hydrogen bonds to form a dimer-like arrangement. These dimers are linked via a C-H···Cl interaction, and the DMF molecule is linked to the complex molecule by C-H···O interactions (Table 1).

### Experimental

A colourless solution of 6,6'-dimethoxy-2,2'-[ethane-1,2-diylbis(nitrilomethanylylidene)]diphenol (54.5 mg,0.2 mmol) in DMF (3 ml) was slowly added to a solution of CoCl_2_ (64 mg, 0.2 mmol) in CH_3_CN(15 ml), forming a dark red solution that was stirred for 30 min at room temperature. Slow evaporation of the solvent at room temperature gave red block-like crystals of the title compound, suitable for X-ray analysis. The crystals were collected by filtration, washed with cold acetonitrile, and dried under vacuum (yield 77%).

### Refinement

The water H atoms were located in an difference electron-density map and allowed to ride on the O atom with O-H = 0.96 Å. The C-bound H-atoms were included in calculated positions and treated as riding atoms: C-H = 0.93, 0.97 and 0.96 Å for CH, CH_2_, and CH_3_ H-atoms, respectively, with U_iso_(H) = k × U_eq_(O,C), where k = 1.5 for OH and CH_3_ H-atoms, and k = 1.2 for other H-atoms.

### Crystal data

**Table:**

[Co(C_18_H_18_N_2_O_4_)Cl(H_2_O)]·C_3_H_7_NO	*F*(000) = 1064
*M**_r_* = 511.84	*D*_x_ = 1.478 Mg m^−^^3^
Monoclinic, *P*2_1_/*c*	Mo *K*α radiation, λ = 0.71073 Å
Hall symbol: -P 2ybc	Cell parameters from 2594 reflections
*a* = 13.1384 (13) Å	θ = 2.3–22.5°
*b* = 13.3144 (19) Å	µ = 0.90 mm^−^^1^
*c* = 14.0120 (9) Å	*T* = 293 K
β = 110.198 (6)°	Block, red
*V* = 2300.4 (4) Å^3^	0.24 × 0.22 × 0.20 mm
*Z* = 4	

### Data collection

**Table:**

Bruker APEXII CCD diffractometer	4034 independent reflections
Radiation source: fine-focus sealed tube	2827 reflections with *I* > 2σ(*I*)
Graphite monochromator	*R*_int_ = 0.087
φ and ω scans	θ_max_ = 25.0°, θ_min_ = 3.0°
Absorption correction: multi-scan (*SADABS*; Sheldrick, 2003)	*h* = −15→15
*T*_min_ = 0.812, *T*_max_ = 0.840	*k* = −15→14
12275 measured reflections	*l* = −16→16

### Refinement

**Table:**

Refinement on *F*^2^	Primary atom site location: structure-invariant direct methods
Least-squares matrix: full	Secondary atom site location: difference Fourier map
*R*[*F*^2^ > 2σ(*F*^2^)] = 0.058	Hydrogen site location: inferred from neighbouring sites
*wR*(*F*^2^) = 0.140	H-atom parameters constrained
*S* = 1.01	*w* = 1/[σ^2^(*F*_o_^2^) + (0.0689*P*)^2^] where *P* = (*F*_o_^2^ + 2*F*_c_^2^)/3
4034 reflections	(Δ/σ)_max_ = 0.001
293 parameters	Δρ_max_ = 0.70 e Å^−^^3^
0 restraints	Δρ_min_ = −0.39 e Å^−^^3^

### Special details

**Table:**

Geometry. Bond distances, angles etc. have been calculated using the rounded fractional coordinates. All su's are estimated from the variances of the (full) variance-covariance matrix. The cell esds are taken into account in the estimation of distances, angles and torsion angles
Refinement. Refinement of *F*^2^ against ALL reflections. The weighted *R*-factor *wR* and goodness of fit *S* are based on *F*^2^, conventional *R*-factors *R* are based on *F*, with *F* set to zero for negative *F*^2^. The threshold expression of *F*^2^ > σ(*F*^2^) is used only for calculating *R*-factors(gt) *etc*. and is not relevant to the choice of reflections for refinement. *R*-factors based on *F*^2^ are statistically about twice as large as those based on *F*, and *R*- factors based on ALL data will be even larger.

### Fractional atomic coordinates and isotropic or equivalent isotropic displacement parameters (Å^2^)

**Table:**

	*x*	*y*	*z*	*U*_iso_*/*U*_eq_	
Co1	0.90848 (4)	0.14998 (3)	0.92275 (3)	0.0496 (2)	
Cl5	0.92093 (9)	0.30523 (7)	0.98338 (7)	0.0715 (4)	
O1	0.90866 (18)	0.09840 (17)	1.04805 (16)	0.0522 (8)	
O2	1.06060 (18)	0.13508 (17)	0.97448 (17)	0.0539 (8)	
O3	0.94754 (18)	0.02362 (19)	1.22469 (16)	0.0611 (9)	
O4	1.26159 (19)	0.1281 (2)	1.08943 (19)	0.0673 (10)	
O5	0.89466 (17)	0.01086 (16)	0.86955 (15)	0.0533 (8)	
N1	0.9079 (2)	0.1957 (2)	0.7953 (2)	0.0535 (10)	
N2	0.7567 (2)	0.1638 (2)	0.8674 (2)	0.0569 (10)	
C1	1.1284 (3)	0.1773 (2)	0.9372 (3)	0.0515 (11)	
C2	1.2396 (3)	0.1745 (3)	0.9968 (3)	0.0606 (14)	
C3	1.3174 (3)	0.2134 (3)	0.9619 (3)	0.0751 (17)	
C4	1.2861 (4)	0.2591 (4)	0.8665 (4)	0.0822 (17)	
C5	1.1799 (4)	0.2652 (3)	0.8074 (3)	0.0737 (17)	
C6	1.0988 (3)	0.2252 (3)	0.8418 (3)	0.0578 (12)	
C7	0.9889 (3)	0.2273 (3)	0.7741 (3)	0.0594 (14)	
C8	0.7993 (3)	0.1895 (3)	0.7181 (3)	0.0698 (16)	
C9	0.7199 (3)	0.2143 (3)	0.7682 (3)	0.0743 (16)	
C10	0.6880 (3)	0.1424 (3)	0.9111 (3)	0.0586 (12)	
C11	0.7150 (3)	0.1020 (3)	1.0106 (3)	0.0541 (12)	
C12	0.6303 (3)	0.0808 (3)	1.0478 (3)	0.0663 (14)	
C13	0.6511 (3)	0.0418 (3)	1.1417 (3)	0.0727 (17)	
C14	0.7565 (3)	0.0214 (3)	1.2036 (3)	0.0665 (16)	
C15	0.8411 (3)	0.0409 (3)	1.1708 (2)	0.0543 (11)	
C16	0.8231 (3)	0.0820 (2)	1.0729 (2)	0.0499 (11)	
C17	0.9757 (3)	−0.0122 (3)	1.3268 (2)	0.0700 (16)	
C18	1.3706 (3)	0.1101 (4)	1.1502 (3)	0.0778 (17)	
O6	0.5199 (4)	0.9040 (5)	0.2962 (4)	0.192 (3)	
N3	0.6662 (5)	0.9626 (6)	0.4178 (4)	0.134 (3)	
C19	0.7766 (5)	0.9485 (6)	0.4778 (4)	0.168 (4)	
C20	0.6114 (8)	1.0463 (11)	0.4401 (8)	0.236 (8)	
C21	0.6116 (6)	0.9002 (6)	0.3479 (5)	0.141 (3)	
H3	1.39040	0.20920	1.00180	0.0900*	
H4	1.33860	0.28570	0.84310	0.0980*	
H5	1.16020	0.29590	0.74400	0.0890*	
H5A	0.87980	−0.03370	0.91700	0.0800*	
H5B	0.96130	−0.00860	0.86080	0.0800*	
H7	0.97560	0.25380	0.70950	0.0710*	
H8A	0.78620	0.12230	0.68970	0.0840*	
H8B	0.79280	0.23660	0.66340	0.0840*	
H9A	0.71680	0.28640	0.77700	0.0890*	
H9B	0.64820	0.19090	0.72730	0.0890*	
H10	0.61510	0.15420	0.87490	0.0700*	
H12	0.55890	0.09390	1.00710	0.0790*	
H13	0.59420	0.02860	1.16480	0.0870*	
H14	0.76990	−0.00570	1.26800	0.0800*	
H17A	0.95880	0.03830	1.36800	0.0840*	
H17B	1.05190	−0.02680	1.35360	0.0840*	
H17C	0.93540	−0.07210	1.32750	0.0840*	
H18A	1.40490	0.07070	1.11250	0.0940*	
H18B	1.37300	0.07430	1.21040	0.0940*	
H18C	1.40800	0.17290	1.16890	0.0940*	
H19A	0.80250	0.88730	0.45800	0.2020*	
H19B	0.78360	0.94460	0.54820	0.2020*	
H19C	0.81850	1.00400	0.46800	0.2020*	
H20A	0.53720	1.04620	0.39530	0.3540*	
H20B	0.64610	1.10710	0.43080	0.3540*	
H20C	0.61390	1.04200	0.50920	0.3540*	
H21	0.65020	0.84530	0.33730	0.1690*	

### Atomic displacement parameters (Å^2^)

**Table:**

	*U*^11^	*U*^22^	*U*^33^	*U*^12^	*U*^13^	*U*^23^
Co1	0.0578 (3)	0.0354 (3)	0.0523 (3)	0.0018 (2)	0.0148 (2)	0.0031 (2)
Cl5	0.0952 (8)	0.0478 (5)	0.0697 (6)	0.0027 (5)	0.0262 (5)	−0.0019 (4)
O1	0.0540 (13)	0.0457 (14)	0.0556 (13)	0.0024 (11)	0.0173 (11)	0.0083 (10)
O2	0.0593 (14)	0.0420 (13)	0.0586 (14)	−0.0011 (11)	0.0179 (11)	0.0085 (10)
O3	0.0649 (16)	0.0616 (16)	0.0533 (14)	0.0079 (13)	0.0158 (12)	0.0069 (11)
O4	0.0587 (16)	0.0688 (18)	0.0702 (16)	0.0024 (13)	0.0171 (13)	0.0054 (13)
O5	0.0599 (14)	0.0371 (12)	0.0615 (13)	−0.0006 (11)	0.0192 (11)	0.0021 (10)
N1	0.0674 (19)	0.0372 (16)	0.0520 (16)	−0.0020 (14)	0.0157 (15)	0.0019 (12)
N2	0.0667 (19)	0.0432 (17)	0.0551 (17)	0.0056 (14)	0.0136 (16)	0.0072 (13)
C1	0.064 (2)	0.0339 (18)	0.060 (2)	−0.0031 (16)	0.0259 (19)	−0.0050 (15)
C2	0.069 (3)	0.050 (2)	0.067 (2)	−0.0035 (19)	0.029 (2)	−0.0060 (17)
C3	0.072 (3)	0.070 (3)	0.087 (3)	−0.008 (2)	0.032 (2)	−0.006 (2)
C4	0.083 (3)	0.082 (3)	0.095 (3)	−0.016 (3)	0.048 (3)	−0.003 (3)
C5	0.097 (3)	0.059 (3)	0.074 (3)	−0.012 (2)	0.041 (3)	0.0032 (19)
C6	0.070 (2)	0.044 (2)	0.062 (2)	−0.0035 (18)	0.026 (2)	−0.0028 (16)
C7	0.086 (3)	0.041 (2)	0.052 (2)	0.0026 (19)	0.025 (2)	0.0048 (15)
C8	0.082 (3)	0.066 (3)	0.052 (2)	−0.002 (2)	0.011 (2)	0.0086 (17)
C9	0.069 (3)	0.074 (3)	0.067 (2)	0.006 (2)	0.007 (2)	0.017 (2)
C10	0.055 (2)	0.046 (2)	0.066 (2)	0.0043 (17)	0.0098 (19)	0.0027 (16)
C11	0.056 (2)	0.044 (2)	0.059 (2)	0.0013 (17)	0.0158 (17)	−0.0028 (15)
C12	0.056 (2)	0.069 (3)	0.072 (2)	0.0048 (19)	0.0197 (19)	−0.002 (2)
C13	0.070 (3)	0.080 (3)	0.075 (3)	0.001 (2)	0.034 (2)	−0.001 (2)
C14	0.077 (3)	0.066 (3)	0.060 (2)	0.002 (2)	0.028 (2)	0.0043 (18)
C15	0.064 (2)	0.0418 (19)	0.056 (2)	0.0031 (17)	0.0192 (18)	−0.0021 (15)
C16	0.060 (2)	0.0353 (18)	0.056 (2)	0.0043 (16)	0.0222 (17)	−0.0025 (14)
C17	0.083 (3)	0.067 (3)	0.054 (2)	0.010 (2)	0.016 (2)	0.0007 (18)
C18	0.061 (3)	0.076 (3)	0.086 (3)	0.004 (2)	0.012 (2)	−0.002 (2)
O6	0.128 (4)	0.242 (7)	0.143 (4)	−0.093 (4)	−0.033 (3)	0.061 (4)
N3	0.121 (4)	0.159 (6)	0.096 (3)	−0.056 (4)	0.005 (3)	0.032 (3)
C19	0.148 (6)	0.189 (8)	0.115 (4)	−0.095 (6)	−0.021 (4)	0.057 (5)
C20	0.215 (11)	0.292 (18)	0.224 (11)	−0.008 (10)	0.105 (8)	−0.032 (11)
C21	0.138 (6)	0.134 (6)	0.103 (4)	−0.075 (5)	−0.019 (4)	0.041 (4)

### Geometric parameters (Å, º)

**Table:**

Co1—Cl5	2.2193 (11)	C11—C12	1.411 (6)
Co1—O1	1.885 (2)	C11—C16	1.412 (5)
Co1—O2	1.887 (3)	C12—C13	1.352 (6)
Co1—O5	1.982 (2)	C13—C14	1.384 (6)
Co1—N1	1.884 (3)	C14—C15	1.366 (6)
Co1—N2	1.883 (3)	C15—C16	1.419 (4)
O1—C16	1.305 (5)	C3—H3	0.9300
O2—C1	1.305 (5)	C4—H4	0.9300
O3—C15	1.360 (4)	C5—H5	0.9300
O3—C17	1.430 (4)	C7—H7	0.9300
O4—C2	1.375 (5)	C8—H8B	0.9700
O4—C18	1.411 (5)	C8—H8A	0.9700
O5—H5A	0.9600	C9—H9B	0.9700
O5—H5B	0.9600	C9—H9A	0.9700
O6—C21	1.173 (9)	C10—H10	0.9300
N1—C7	1.271 (5)	C12—H12	0.9300
N1—C8	1.465 (5)	C13—H13	0.9300
N2—C10	1.286 (5)	C14—H14	0.9300
N2—C9	1.468 (5)	C17—H17A	0.9600
N3—C19	1.416 (9)	C17—H17B	0.9600
N3—C20	1.419 (15)	C17—H17C	0.9600
N3—C21	1.296 (10)	C18—H18B	0.9600
C1—C6	1.409 (5)	C18—H18C	0.9600
C1—C2	1.411 (6)	C18—H18A	0.9600
C2—C3	1.378 (6)	C19—H19A	0.9600
C3—C4	1.395 (7)	C19—H19B	0.9600
C4—C5	1.357 (7)	C19—H19C	0.9600
C5—C6	1.416 (7)	C20—H20A	0.9600
C6—C7	1.428 (6)	C20—H20B	0.9600
C8—C9	1.482 (6)	C20—H20C	0.9600
C10—C11	1.420 (6)	C21—H21	0.9300
			
Cl5—Co1—O1	90.38 (7)	C11—C16—C15	117.8 (4)
Cl5—Co1—O2	91.29 (8)	O1—C16—C15	116.8 (3)
Cl5—Co1—O5	178.77 (8)	O1—C16—C11	125.4 (3)
Cl5—Co1—N1	92.15 (9)	C2—C3—H3	120.00
Cl5—Co1—N2	89.67 (9)	C4—C3—H3	120.00
O1—Co1—O2	86.75 (10)	C5—C4—H4	120.00
O1—Co1—O5	88.87 (9)	C3—C4—H4	120.00
O1—Co1—N1	177.47 (11)	C4—C5—H5	120.00
O1—Co1—N2	94.58 (11)	C6—C5—H5	120.00
O2—Co1—O5	89.63 (10)	N1—C7—H7	118.00
O2—Co1—N1	93.16 (11)	C6—C7—H7	117.00
O2—Co1—N2	178.35 (11)	C9—C8—H8B	110.00
O5—Co1—N1	88.60 (10)	H8A—C8—H8B	108.00
O5—Co1—N2	89.42 (11)	C9—C8—H8A	110.00
N1—Co1—N2	85.46 (12)	N1—C8—H8A	110.00
Co1—O1—C16	125.8 (2)	N1—C8—H8B	110.00
Co1—O2—C1	125.4 (2)	N2—C9—H9A	110.00
C15—O3—C17	118.2 (3)	N2—C9—H9B	110.00
C2—O4—C18	119.0 (3)	C8—C9—H9A	110.00
Co1—O5—H5A	109.00	C8—C9—H9B	110.00
Co1—O5—H5B	109.00	H9A—C9—H9B	109.00
H5A—O5—H5B	110.00	C11—C10—H10	117.00
Co1—N1—C7	126.8 (3)	N2—C10—H10	118.00
Co1—N1—C8	111.5 (2)	C11—C12—H12	120.00
C7—N1—C8	121.8 (3)	C13—C12—H12	119.00
C9—N2—C10	119.8 (3)	C12—C13—H13	120.00
Co1—N2—C10	126.9 (3)	C14—C13—H13	120.00
Co1—N2—C9	113.0 (2)	C15—C14—H14	120.00
C19—N3—C21	123.4 (7)	C13—C14—H14	120.00
C20—N3—C21	118.7 (8)	O3—C17—H17B	109.00
C19—N3—C20	117.9 (7)	H17A—C17—H17C	109.00
C2—C1—C6	117.6 (4)	O3—C17—H17C	110.00
O2—C1—C2	117.5 (3)	H17A—C17—H17B	109.00
O2—C1—C6	124.9 (4)	O3—C17—H17A	110.00
O4—C2—C1	114.1 (3)	H17B—C17—H17C	109.00
O4—C2—C3	124.4 (4)	O4—C18—H18C	110.00
C1—C2—C3	121.6 (4)	H18A—C18—H18C	109.00
C2—C3—C4	119.7 (4)	H18B—C18—H18C	110.00
C3—C4—C5	120.8 (5)	H18A—C18—H18B	109.00
C4—C5—C6	120.4 (4)	O4—C18—H18A	109.00
C5—C6—C7	118.3 (4)	O4—C18—H18B	109.00
C1—C6—C5	120.0 (4)	O6—C21—N3	128.4 (8)
C1—C6—C7	121.6 (4)	N3—C19—H19A	109.00
N1—C7—C6	125.1 (4)	N3—C19—H19B	109.00
N1—C8—C9	107.6 (3)	N3—C19—H19C	110.00
N2—C9—C8	106.9 (3)	H19A—C19—H19B	109.00
N2—C10—C11	125.0 (4)	H19A—C19—H19C	110.00
C12—C11—C16	119.2 (3)	H19B—C19—H19C	109.00
C10—C11—C16	122.4 (4)	N3—C20—H20A	109.00
C10—C11—C12	118.5 (4)	N3—C20—H20B	109.00
C11—C12—C13	121.1 (4)	N3—C20—H20C	109.00
C12—C13—C14	120.5 (4)	H20A—C20—H20B	110.00
C13—C14—C15	120.4 (4)	H20A—C20—H20C	109.00
O3—C15—C14	125.5 (3)	H20B—C20—H20C	110.00
C14—C15—C16	121.0 (3)	O6—C21—H21	116.00
O3—C15—C16	113.5 (3)	N3—C21—H21	116.00
			
Cl5—Co1—O1—C16	90.4 (2)	Co1—N2—C10—C11	−1.2 (6)
O2—Co1—O1—C16	−178.3 (2)	C10—N2—C9—C8	−155.7 (3)
O5—Co1—O1—C16	−88.6 (2)	C19—N3—C21—O6	178.1 (8)
N2—Co1—O1—C16	0.7 (2)	C20—N3—C21—O6	2.1 (13)
Cl5—Co1—O2—C1	−73.5 (2)	O2—C1—C6—C5	−177.6 (3)
O1—Co1—O2—C1	−163.8 (3)	C2—C1—C6—C7	176.8 (4)
O5—Co1—O2—C1	107.3 (3)	O2—C1—C2—O4	−1.3 (5)
N1—Co1—O2—C1	18.7 (3)	O2—C1—C6—C7	−3.0 (6)
Cl5—Co1—N1—C7	75.7 (3)	O2—C1—C2—C3	177.3 (3)
Cl5—Co1—N1—C8	−105.3 (2)	C2—C1—C6—C5	2.2 (5)
O2—Co1—N1—C7	−15.8 (3)	C6—C1—C2—C3	−2.6 (5)
O2—Co1—N1—C8	163.3 (2)	C6—C1—C2—O4	178.9 (3)
O5—Co1—N1—C7	−105.3 (3)	C1—C2—C3—C4	1.7 (6)
O5—Co1—N1—C8	73.7 (2)	O4—C2—C3—C4	−180.0 (4)
N2—Co1—N1—C7	165.2 (3)	C2—C3—C4—C5	−0.4 (7)
N2—Co1—N1—C8	−15.8 (2)	C3—C4—C5—C6	0.1 (7)
Cl5—Co1—N2—C9	83.4 (2)	C4—C5—C6—C7	−175.8 (4)
Cl5—Co1—N2—C10	−89.9 (3)	C4—C5—C6—C1	−1.0 (6)
O1—Co1—N2—C9	173.8 (2)	C5—C6—C7—N1	−178.8 (4)
O1—Co1—N2—C10	0.5 (3)	C1—C6—C7—N1	6.6 (6)
O5—Co1—N2—C9	−97.4 (2)	N1—C8—C9—N2	−41.8 (4)
O5—Co1—N2—C10	89.3 (3)	N2—C10—C11—C16	0.7 (6)
N1—Co1—N2—C9	−8.8 (2)	N2—C10—C11—C12	−179.0 (4)
N1—Co1—N2—C10	177.9 (3)	C10—C11—C12—C13	179.7 (4)
Co1—O1—C16—C11	−1.3 (4)	C16—C11—C12—C13	0.0 (6)
Co1—O1—C16—C15	178.8 (2)	C12—C11—C16—C15	0.1 (5)
Co1—O2—C1—C2	167.5 (2)	C12—C11—C16—O1	−179.7 (3)
Co1—O2—C1—C6	−12.7 (5)	C10—C11—C16—O1	0.6 (5)
C17—O3—C15—C16	176.4 (3)	C10—C11—C16—C15	−179.5 (3)
C17—O3—C15—C14	−4.1 (5)	C11—C12—C13—C14	−0.2 (6)
C18—O4—C2—C3	−6.1 (6)	C12—C13—C14—C15	0.3 (6)
C18—O4—C2—C1	172.4 (3)	C13—C14—C15—C16	−0.2 (6)
Co1—N1—C8—C9	36.3 (3)	C13—C14—C15—O3	−179.7 (4)
C7—N1—C8—C9	−144.6 (4)	O3—C15—C16—C11	179.6 (3)
Co1—N1—C7—C6	6.2 (6)	C14—C15—C16—O1	179.8 (3)
C8—N1—C7—C6	−172.7 (4)	C14—C15—C16—C11	0.0 (5)
Co1—N2—C9—C8	30.5 (4)	O3—C15—C16—O1	−0.6 (4)
C9—N2—C10—C11	−174.1 (4)		

### Hydrogen-bond geometry (Å, º)

**Table:**

*D*—H···*A*	*D*—H	H···*A*	*D*···*A*	*D*—H···*A*
O5—H5*A*···O2^i^	0.96	1.98	2.830 (3)	146
O5—H5*A*···O4^i^	0.96	2.22	2.964 (4)	134
O5—H5*B*···O1^i^	0.96	2.11	2.840 (3)	131
O5—H5*B*···O3^i^	0.96	1.97	2.854 (3)	151
C9—H9*B*···O6^ii^	0.97	2.47	3.355 (7)	152
C10—H10···O6^ii^	0.93	2.56	3.287 (7)	135
C17—H17*A*···Cl5^iii^	0.96	2.79	3.744 (4)	175

Symmetry codes: (i) −*x*+2, −*y*, −*z*+2; (ii) −*x*+1, −*y*+1, −*z*+1; (iii) *x*, −*y*+1/2, *z*+1/2.
